# Bioactive and Functional Properties of Protein Isolates and Hydrolysates from Edible Insects

**DOI:** 10.3390/foods15152729

**Published:** 2026-08-03

**Authors:** Danka Dragojlović, Ivana Čabarkapa, Dragana Tomanić, Tea Sedlar, Jelena Vujetić, Olivera Đuragić, Ljiljana Popović

**Affiliations:** 1 Institute of Food Technology in Novi Sad, University of Novi Sad, Bulevar Cara Lazara 1, 21000 Novi Sad, Serbia; danka.dragojlovic@fins.uns.ac.rs (D.D.); dragana.tomanic@fins.uns.ac.rs (D.T.); tea.sedlar@fins.uns.ac.rs (T.S.); jelena.vujetic@fins.uns.ac.rs (J.V.); olivera.djuragic@fins.uns.ac.rs (O.Đ.); 2 Faculty of Technology Novi Sad, University of Novi Sad, Bulevar Cara Lazara 1, 21000 Novi Sad, Serbia; ljiljana04@tf.uns.ac.rs

**Keywords:** edible insects, bioactive proteins and peptides, antimicrobial activity, antioxidative activity, techno-functional characteristics

## Abstract

This study investigated the isolation and bioactive characterization of proteins obtained from insect flours of *Tenebrio molitor*, *Zophobas morio*, and *Acheta domesticus*. Protein isolates were produced from defatted insect flours using a modified alkaline extraction procedure, including extraction at pH 11, isoelectric precipitation at pH 4, and an additional purification step aimed at improving protein purity and isolate quality. The obtained protein isolates were characterized in terms of chemical composition, electrophoretic profile, solubility, water- and oil-holding capacities, amino acid composition, and antioxidant activity. All isolates exhibited notable antioxidant potential in the 2,2-azino-bis-3-ethylbenzothiazoline-6-sulfonic acid (ABTS) assay, indicating their ability to act as natural radical-scavenging compounds. In order to further evaluate their bioactive potential, the protein isolates were subjected to enzymatic hydrolysis using Alcalase. The obtained protein hydrolysates exhibited antimicrobial activity against both *Escherichia coli* and *Staphylococcus epidermidis*, whereas the corresponding protein isolates showed no antimicrobial activity under the applied conditions. Among the investigated samples, *Zophobas morio* hydrolysate showed the highest antioxidant potential and degree of hydrolysis, whereas *Acheta domesticus* hydrolysate demonstrated the strongest antibacterial activity. These findings highlight the potential of edible insect-derived proteins as sustainable sources of natural antioxidant and antimicrobial ingredients for future applications in the food, feed, pharmaceutical, and cosmetic industries.

## 1. Introduction

Edible insects are increasingly investigated as sustainable sources of high-quality proteins and as promising ingredients for functional food and nutraceutical applications. Among them, yellow mealworm (*Tenebrio molitor*, TM), house cricket (*Acheta domesticus*, AD) and superworm (*Zophobas morio*, ZM) are of particular interest due to their relatively high protein content, favourable amino acid composition and potential for processing into flours, protein concentrates, isolates and hydrolysates [1,2,3]. Processed insect ingredients are also considered more acceptable to consumers than whole insects, which may support their wider use in food systems [3]. From a technological perspective, insect protein isolates (PI) are valuable not only due to their nutritional quality but also because of their techno-functional properties, including solubility, water- and oil-holding capacity, foaming, emulsifying and gel-forming ability. These properties determine their potential application in protein-enriched foods, bakery products, meat analogues, emulsified systems, beverages and functional snacks [3,4,5]. Protein solubility is particularly important because it strongly influences other functional properties and depends on pH, protein structure, molecular size, extraction conditions and processing history [3]. In addition to their functional role, insect proteins are increasingly recognized as precursors of bioactive peptides. Recent studies have shown that edible insect protein hydrolysates and peptides may exhibit antioxidant, antihypertensive, antidiabetic, anti-inflammatory and antimicrobial activities [6,7,8,9,10,11]. For TM, several studies have reported the potential of protein hydrolysates as sources of bioactive peptides. Rivero-Pino et al. [6] demonstrated that TM protein hydrolysates (PH) obtained with food-grade proteases such as subtilisin, trypsin, ficin and Flavourzyme showed ACE-inhibitory, antioxidant and DPP-IV inhibitory activities. In a later study, Rivero-Pino et al. [11] identified DPP-IV and α-glucosidase inhibitory peptide fractions from TM, suggesting their possible use in ingredients aimed at glycaemic control. Tan et al. [7] further identified DPP-4 inhibitory peptides from Flavourzyme hydrolysates of TM proteins, and confirmed their interaction with DPP-4 by molecular docking. These findings indicate that TM proteins are promising precursors of antidiabetic and multifunctional bioactive peptides.

Compared with TM, fewer studies have focused on AD, although recent results indicate strong bioactive potential. Teixeira et al. [8] reported ACE-inhibitory peptides released during in vitro gastrointestinal digestion of *A. domesticus*. Summart et al. [9] identified antioxidant and ACE-inhibitory peptide fractions from AD PH and suggested their possible role in cellular antioxidant protection. In addition, Yeerong et al. [10] optimized Alcalase hydrolysis of AD proteins and reported antioxidant, anti-inflammatory and anti-skin-ageing activities of the obtained hydrolysates. These studies support the potential of AD proteins as sources of bioactive peptides for functional food and nutraceutical applications. The available literature on ZM is more limited, especially regarding sequence-identified peptides. However, recent studies suggest that ZM proteins and hydrolysates may possess antioxidant and anti-inflammatory potential. Pečová et al. [12] reported that enzymatically hydrolysed ZM samples showed antioxidant and anti-inflammatory effects influenced by rearing substrate. More recent studies on ZM protein extraction and hydrolysis have also indicated that processing conditions, including ultrasound-assisted hydrolysis and alternative extraction approaches, may improve protein recovery, modify structural characteristics and enhance antioxidant or anti-inflammatory properties [13,14]. Nevertheless, compared with TM and AD, peptide identification and mechanism-oriented studies on ZM remain scarce.

The bioactive potential of insect-derived protein hydrolysates depends on several factors, including insect species, protein fraction, extraction method, enzyme specificity, degree of hydrolysis, molecular weight distribution and amino acid sequence of released peptides [2,3,6]. Enzymes such as Alcalase, Flavourzyme, Protamex, Neutrase, Pepsin and Trypsin are commonly used to generate insect protein hydrolysates with improved biological activity [3,6,7,15]. In general, the literature data suggest that low-molecular-weight peptides may often be associated with higher bioactivity due to better accessibility to biological targets and possible absorption during digestion. However, such effects are strongly dependent on peptide sequence, composition and structure.

Protein extraction is also an important step affecting the quality, functionality and biological potential of insect-derived ingredients. Alkaline extraction followed by isoelectric precipitation remains one of the most widely used approaches for obtaining insect PIs because it is relatively simple, cost-effective and applicable to different insect flours. Raw material preparation usually includes drying, grinding and defatting, since the relatively high lipid content of many insects can influence protein extraction yield, colour, flavour, oxidative stability and techno-functional behavior [3,16,17]. Although emerging technologies such as ultrasound-assisted extraction, microwave-assisted extraction, membrane separation and green solvent-based methods have recently been investigated, alkaline extraction remains a practical approach for producing protein isolates intended for functional and bioactive characterization [13,14,18]. Despite increasing interest in edible insect proteins, comparative studies evaluating both functional properties and biological activities of PIs and corresponding hydrolysates from different insect species remain limited. This is particularly evident for ZM, for which fewer sequence-resolved bioactive peptide studies are available compared with TM and AD. Therefore, direct comparison of these species under similar extraction, hydrolysis and analytical conditions may provide useful information about their potential as techno-functional and bioactive ingredients.

Therefore, the aim of this study was to isolate proteins from three edible insect flours: TM, ZM and AD, and to evaluate their functional and bioactive potential. The obtained PIs were characterized in terms of proximate composition, amino acid profile, pH-dependent solubility and SDS-PAGE electrophoretic patterns. In addition, their techno-functional properties and selected biological activities were determined. The study also aimed to assess the potential of the corresponding hydrolysates as sources of bioactive peptides, providing comparative insight into the suitability of TM, ZM and AD proteins as functional and bioactive ingredients for future food and nutraceutical applications.

## 2. Materials and Methods

### 2.1. Insect Rearing and Sample Preparation

TM, ZM and AD were reared at the Institute of Food Technology in Novi Sad. Wheat bran was used as the main substrate, while vegetables were provided as a water source. After harvesting, the insects were starved for 24 h to empty the digestive tract and then inactivated by blanching in boiling water for 30 s. The samples were dried in a vacuum oven (Binder GmbH, Tuttlingen, Germany) at 50 °C for 8 h. Dried insects were ground using a cooled laboratory mill (KN 295 Knifetec™, FOSS, Hillerød, Denmark); defatted using n-hexane at a flour-to-solvent ratio of 1:5; and stored frozen until further analysis.

### 2.2. Protein Isolation

Protein isolation from TM, ZM, and AD flours was performed as a modification of the alkaline extraction method described by Azagoh et al. [17]. Protein extraction was carried out in an aqueous solution at pH 11, adjusted with 1 M NaOH. The extraction process was performed under constant stirring for 1 h at room temperature. After extraction, the protein suspension was centrifuged at 10,000 rpm for 20 min at 4 °C using a Sorvall^®^ RC-5B Refrigerated Superspeed Centrifuge (Du Pont Instruments, Wilmington, DE, USA), and the supernatant was collected. Protein precipitation was achieved by adjusting the pH of the supernatant to 4 using 1 M HCl under continuous stirring, resulting in the formation of a whitish precipitate. The suspension was stored overnight at 4 °C to allow complete precipitation and subsequently centrifuged again at 10,000 rpm for 20 min at 4 °C. For further purification, the extraction and precipitation procedure was repeated once more under the same conditions. After the second precipitation step, the obtained protein precipitate was dried overnight in a drying oven (Binder FD56, Tuttlingen, Germany) at 30 °C and ground into powder using a laboratory mortar and pestle.

### 2.3. Proximate Analysis

Crude protein content was determined using the Kjeldahl method [19]. A nitrogen-to-protein conversion factor of 5.6 was used because the analysed samples were purified protein isolates rather than whole-insect flours, and this factor has previously been applied to insect protein fractions obtained by alkaline extraction and purification [18,20,21]. Moisture content was determined according to AOAC method 934.01, crude fat by Soxhlet extraction according to AOAC method 920.39 [22], crude ash according to AOAC method 942.05 [23]. All analyses were performed in triplicate, and the results were expressed on a dry matter basis.

### 2.4. Amino Acid Analysis

Amino acid composition was determined using ion-exchange chromatography. Prior to analysis, samples were hydrolyzed in 6 M HCl at 110 °C for 24 h. To prevent amino acid degradation during hydrolysis, 1% phenol and 0.5% thioglycolic acid were added as protective agents. After hydrolysis, samples were cooled to room temperature, dissolved in loading buffer (pH 2.2), filtered through a 0.22 μm PTFE filter, and transferred into vials. Amino acid analysis of PIs was performed using a Biochrom 30+ amino acid analyzer (Biochrom, Cambridge, UK) according to the method of Spackman et al. [24]. Following chromatographic separation, post-column derivatization with ninhydrin was performed. Amino acids were detected at 570 nm, while proline was detected at 440 nm. Identification was based on the retention times of amino acid standards, and quantification was performed using calibration curves of the standard solution (Sigma-Aldrich, St. Louis, MO, USA). Results were expressed as g of amino acids per 100 g of protein and presented as the mean value of three replicates.

### 2.5. SDS-PAGE Electrophoresis

SDS-PAGE analysis of the PI and PH was performed according to the method described by Laemmli [25]. The electrophoretic system consisted of a stacking gel containing 4% acrylamide and a resolving gel containing 10% acrylamide. PI samples were dissolved in Tris/glycine buffer (pH 6.8) containing 20 g/L sodium dodecyl sulfate (SDS) and 50 g/L β-mercaptoethanol. Gels were stained using Coomassie Brilliant Blue R-250 and silver staining. Electrophoresis was carried out using a Multi Drive XL apparatus (Pharmacia, Uppsala, Sweden) at 60 mA until the dye front reached the bottom of the gel plate.

### 2.6. Protein Solubility

Protein solubility was determined at different pH values ranging from 2 to 11 using buffer solutions. Briefly, 10 mg of PI was weighed and mixed with 1 mL of the corresponding buffer solution. The protein suspensions were incubated for 1 h at room temperature using a Biosan TS 100-C shaker (Biosan, Riga, Latvia). After incubation, the samples were centrifuged at 14,500 rpm for 10 min using an Eppendorf Mini Spin Plus centrifuge (Eppendorf, Hamburg, Germany). The concentration of soluble proteins in the supernatant was determined according to the Bradford method [26]. Protein solubility was expressed as the concentration of soluble proteins, expressed as bovine serum albumin (BSA) equivalents, and presented as the mean value of three replicates.

### 2.7. Water and Oil-Holding Capacity (WHC, OHC)

The water- (WHC) and oil-holding capacities (OHC) of the protein isolates were determined according to the methods described by Sedlar et al. [27]. Briefly, 0.1 g of PI was weighed into a 1.5 mL Eppendorf tube, followed by the addition of 1 mL of water or vegetable oil. The tubes were mixed using a Thermo-Shaker TS-100C (Biosan, Latvia) for 30 min at room temperature (25 ± 2 °C) and subsequently centrifuged at 14,500 rpm for 20 min using an Eppendorf Mini Spin Plus centrifuge (Eppendorf, Germany). After centrifugation, the tubes were inverted onto filter paper and left for 30 min to remove excess water or oil. The final mass of the tube and sample was then measured. Water- and oil-holding capacities were expressed as grams of water or oil per gram of PI (g/g) and presented as the mean values of three measurements.

### 2.8. Antioxidant Activity of PI and PH

The antioxidant activity of PIs was determined spectrophotometrically using the ABTS•+ radical cation assay according to the method described by Popović et al. [28]. The assay was based on the discoloration of the green ABTS•+ radical cation solution in the presence of protein isolates, measured at 734 nm. The stock ABTS•+ solution was prepared by dissolving ABTS in 0.1 M phosphate-buffered saline (PBS, pH 7.4) containing 5 mM NaCl. The prepared solution was stored in the dark for 12–24 h to allow the formation of free radicals. On the day of analysis, the solution was diluted with PBS to obtain an absorbance value of 0.7 at 734 nm. Subsequently, 30 μL of the sample was added to 3 mL of the working ABTS•+ solution. The decrease in absorbance was monitored for 10 min together with a blank sample. Antioxidant activity was determined in triplicate, and the results were presented as IC_50_ values.

### 2.9. Determination of Antimicrobial Activity

Antimicrobial activity was determined according to CLSI [29] recommendations, with modifications described by Čabarkapa et al. [30]. The antimicrobial activity of insect protein hydrolysates (PHs) was evaluated against the Gram-negative bacterium *Escherichia coli* and the Gram-positive bacterium *Staphylococcus epidermidis*. Briefly, 100 μL of Mueller–Hinton broth (MHB, HiMedia, Mumbai, India) was added to each well of a 96-well microtiter plate, except for the first well. PHs were serially diluted to obtain final concentrations ranging from 0.71 to 21.36 mg/mL depending on the hydrolysate. Subsequently, 10 μL of bacterial suspension was added to each well to achieve a final bacterial concentration of 10^6^ CFU/mL. Plates were incubated at 37 °C for 24 h. Growth control (MHB + bacteria), sterility control I (MHB + PH), and sterility control II (MHB only) were included. After incubation, 10 μL of 0.01% resazurin solution (Sigma-Aldrich, St. Louis, MO, USA) was added to each well, followed by additional incubation at 37 °C for 6 h in the dark. The minimum inhibitory concentration (MIC) was defined as the lowest PH concentration that prevented the colour change in resazurin from its oxidized to reduced form. MIC values were confirmed by plating 100 μL from each well onto Mueller–Hinton agar (MHA, HiMedia, India), followed by incubation at 37 °C for 24 h. The bactericidal or bacteriostatic effect of PH was additionally evaluated using the time-kill assay according to CLSI [31]. Briefly, 1 mL of PH was inoculated with 100 μL of bacterial suspension containing 10^6^ CFU/mL. The initial bacterial count was determined immediately after inoculation (0 h), while bacterial survival after 24 h was determined by the standard plate count method on Nutrient Agar (NA, HiMedia, India). Results were expressed as log_10_ CFU/mL.

### 2.10. Enzymatic Hydrolysis of PI

PIs were dissolved in glycine buffer at pH 9. The buffer was selected based on the optimal enzyme activity and PI solubility. The prepared solution was continuously stirred and heated to 50 °C. After 1 h of mixing, commercial Alcalase enzyme, protease from *Bacillus licheniformis* (2.4 AU/g, Sigma-Aldrich, St. Louis, MO, USA), was added at an enzyme-to-substrate ratio (E/S) of 1:200 (*w*/*w*). Hydrolysis was carried out for 210 min, and samples were collected at 0, 30, 60, 120, 180, and 210 min to monitor the progress of the reaction. After collection, the samples were thermally inactivated by boiling in a water bath for 5 min. To separate denatured proteins and enzymes from the hydrolysates, the samples were centrifuged using an Eppendorf Mini Spin Plus centrifuge at 14,500 rpm for 5 min.

### 2.11. Determination of Degree of Hydrolysis

The degree of hydrolysis (DH) was determined using the trichloroacetic acid (TCA) method according to Tsumura et al. [32]. During enzymatic hydrolysis, samples were collected and mixed with 0.44 M TCA solution at a ratio of 1:1. The prepared mixtures were stored at 4 °C for 30 min to allow protein precipitation. Subsequently, the concentration of non-precipitated proteins in the supernatant was determined according to the Bradford method [26]. The degree of hydrolysis was calculated using the following Equation (1):

(1) $$DH=\frac{C_{TCA}}{C_{PRO}}\times 100$$

where *DH* is the degree of hydrolysis (%), *C_TCA_* the concentration of proteins in the TCA-soluble fraction (mg/mL), and *C_PRO_* is the concentration of total proteins (mg/mL).

### 2.12. Statistical Analysis

The data were processed statistically using the software package XLSTAT 2024 (Lumivero, Denver, CO, USA). Results were expressed as mean ± standard deviation. Analysis of variance (ANOVA) and Tukey’s HSD test (α = 0.05) were used for comparison of sample means.

## 3. Results and Discussion

### 3.1. Chemical Composition

The chemical composition of insect protein isolates obtained after double alkaline extraction is presented in Table 1.

The crude protein content obtained in the present study was compared with previously reported data on PIs and concentrates from edible insects. The protein content of TM PI (74.98%) was in very close agreement with the value reported by Yi et al. [33], who obtained a TM PI containing 74% protein after aqueous/alkaline extraction and acid precipitation at pH 4. This indicates that the double alkaline extraction procedure applied in the present study was effective for TM protein enrichment and produced an isolate with protein purity comparable to previously reported mealworm PI. Furthermore, Pinel et al. [18] reported that mealworm protein concentrates produced by isoelectric precipitation contained approximately 80% protein, whereas concentrates obtained by ultrafiltration/diafiltration contained around 72% protein. Therefore, the protein content of TM PI observed in the present study falls within the range reported for mealworm protein concentrates and isolates obtained by wet extraction techniques. The protein content of AD PI (68.88%) was lower than values reported for highly purified cricket protein isolates. For example, Edward et al. [34] reported that alkaline extraction–acid precipitation combined with 60% ammonium sulfate produced a cricket protein isolate with approximately 94% protein. However, this higher value was achieved using an additional salting-out purification step, which was not applied in the present study. Therefore, the lower protein content of AD PI may be attributed to the absence of additional purification, as well as to species-specific differences in protein solubility, matrix composition, and the co-precipitation of non-protein components. Nevertheless, the obtained value confirms that the applied procedure successfully produced a protein-enriched fraction from house cricket flour. The lowest protein content was observed in ZM PI (65.29%). Previous studies on *Zophobas morio* protein isolation are still limited compared with those on TM and AD. Zhatkanbayeva et al. [35] reported that isoelectric precipitation after alkaline extraction was the most effective method for recovering proteins from *Zophobas morio* larvae, giving a protein yield of 66.09% of the initial dry matter. Although this value refers to protein yield rather than protein content in the final isolate, it supports the suitability of alkaline extraction followed by precipitation for superworm protein recovery.

### 3.2. Amino Acid Composition

The highest total quantified amino acid content was found in the ZM PI (76.55%), whereas the lowest value was determined in the AD PI (44.80%) (Table 2). A similar trend was observed for essential amino acids, with the highest content recorded in the ZM PI (31.99%) and the lowest in the AD PI (16.59%).

These results indicate pronounced species-dependent differences in the amino acid composition of the investigated insect PIs, which is in agreement with previous studies showing that the nutritional composition of edible insects depends on species, developmental stage, diet and processing conditions [1,3,33,36]. Among non-essential amino acids, aspartic acid, glutamic acid and tyrosine were the predominant amino acids. Aspartic and glutamic acids were present in relatively high amounts in all samples, while tyrosine was particularly abundant in the TM and ZM PIs. The high proportion of acidic amino acids may contribute to the nutritional and functional properties of insect PI, especially their solubility and interaction with water, whereas the high tyrosine content may increase the nutritional relevance of these isolates, given the role of tyrosine as a precursor of biologically important compounds [37,38]. The most abundant essential amino acids in the investigated PIs were leucine, lysine, valine and isoleucine. This amino acid pattern confirms the potential of insect PIs as valuable alternative protein sources for food and feed applications. Recent reviews also indicate that edible insects generally contain high-quality proteins with a favourable essential amino acid profile, which may be comparable to conventional protein sources used in human and animal nutrition [36,39]. In particular, the relatively high lysine content is nutritionally important, since lysine is often limiting in cereal-based diets. Therefore, supplementation of cereal-based formulations with insect protein isolates, especially those obtained from ZM and TM could improve their amino acid balance [37]. Similarly, the addition of insect PIs to feed mixtures containing sunflower meal may contribute to improving lysine content, since sunflower meal is known to be relatively deficient in this essential amino acid. Methionine was the limiting essential amino acid in all three PIs, which is in agreement with previous findings indicating that sulphur-containing amino acids may represent a limiting factor in some insect proteins [1,33,36]. In practical food/feed applications, the addition of synthetic methionine or combination with methionine-rich protein sources may be required in order to achieve a balanced amino acid profile. Phenylalanine was not detected in any of the investigated protein isolates. In many studies, tyrosine and phenylalanine are presented as their combined value due to their structural and biological relationship; therefore, direct comparison with literature data should be made carefully [1,33,40]. Moreover, phenylalanine was detected in the standard mixture and in the corresponding whole-insect flours analysed under the same chromatographic conditions, suggesting that its non-detection in the isolates was sample-specific rather than the result of a general analytical or calibration error. In addition, recent studies emphasize that protein extraction conditions may considerably affect protein recovery, composition and final nutritional quality of insect-derived protein ingredients [39]. Compared with commercially available PIs, insect PIs may be considered nutritionally promising due to their favourable essential amino acid composition. The essential amino acid content of TM and ZM PIs is comparable to that reported for soybean proteins, whereas casein generally contains higher levels of essential amino acids than the investigated insect isolates [16]. However, protein quality should not be assessed solely on the basis of amino acid composition. Digestibility, bioavailability of indispensable amino acids and processing conditions are also crucial for determining the real nutritional value of insect proteins. In this regard, Malla et al. [36] emphasized that comparable evaluation of insect protein quality requires the use of appropriate assessment methods, including amino acid digestibility-based approaches such as DIAAS. In addition, recent research has shown that culinary and technological treatments can significantly influence the digestibility and protein quality of edible insects, including TM and cricket species [41]. Overall, the results obtained in this study indicate that PIs from TM, ZM and AD represent promising alternative protein ingredients. Among them, the ZM PI showed the most favourable amino acid profile, particularly due to its highest total quantified amino acid and essential amino acid contents. However, the relatively low methionine content and the absence of detectable phenylalanine indicate that further optimisation of extraction conditions and additional nutritional evaluation, including digestibility studies, are necessary before these isolates can be fully recommended for broader food and feed applications.

### 3.3. SDS Gel Electrophoresis of PI

**Figure 1:**
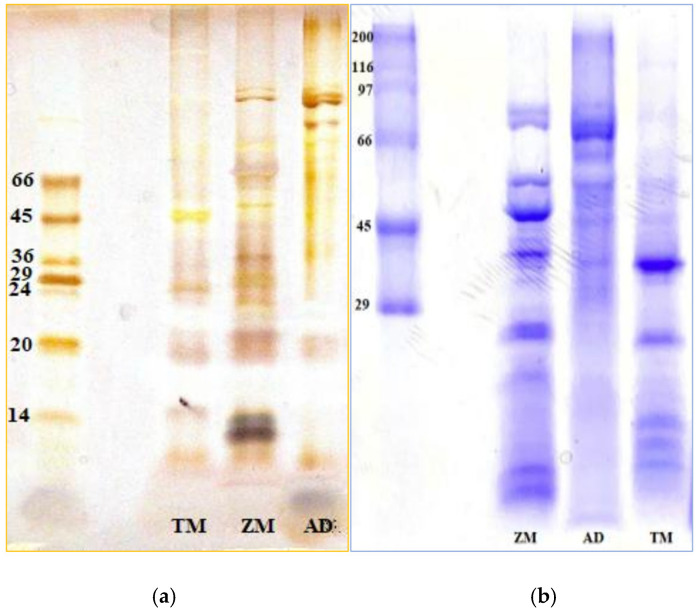
Electrophoretic profile of *Tenebrio molitor* (TM), *Zophobas morio* (ZM) and *Acheta domesticus* (AD) protein isolates in the molecular weight ranges of 14–66 kDa (silver stained gel) (**a**) and 29–200 kDa (Coomassie Brilliant Blue R-250) (**b**).

Separation of protein fractions by SDS-PAGE resulted in the formation of protein bands covering a wide range of molecular weights (Figure 1).

Since the molecular weight distribution was broad, two molecular weight standards were used to estimate and compare both low-molecular-weight (Figure 1a) and high-molecular-weight protein fractions (Figure 1b). SDS-PAGE is commonly used for the characterization of edible insect proteins, since it enables the separation of proteins according to their molecular weight. However, the exact identification of individual insect proteins remains challenging because complete proteomic databases are still not available for many edible insect species. Therefore, the assignment of protein bands is usually based on comparison with previously reported molecular weights and available proteomic data [3]. Accordingly, the band assignments discussed below should be considered tentative and based on molecular weight similarities with literature data, rather than on direct experimental protein identification.

In lane 1, molecular weight markers ranging from 14 to 66 kDa (Figure 1a) and from 29 to 200 kDa (Figure 1b) can be observed. In all PI samples, distinct bands below 14 kDa and around 20 kDa were clearly visible. The electrophoretic profiles of the TM and ZM PIs were similar, whereas both differed from the profile of the cricket PI. This similarity may be associated with the taxonomic relatedness of TM and ZM, since both species belong to the family Tenebrionidae. In addition, differences between the AD isolate and the two larval isolates may be related to species-specific protein composition, developmental stage, tissue composition and the extraction behaviour of soluble and insoluble protein fractions.

In the TM PI, five intense bands could be observed. The first band was located below 14 kDa, the second around 14 kDa, the third around 20 kDa, the fourth between 24 and 29 kDa, and the fifth around 45 kDa. Based on comparison with literature data, proteins with molecular weights below 14 kDa could tentatively correspond to low-molecular-weight hemolymph proteins, which have been reported to have molecular weights of approximately 12 kDa [42]. More recent literature also indicates that proteins below 14 kDa in TM are often associated with hemolymph proteins, including antifreeze-related proteins with molecular weights of approximately 8.5–13 kDa [3]. Among myofibrillar proteins, myosin represents one of the major protein fractions in mealworm. Depending on the sample and protein chain type, myosin-related proteins may occur at approximately 205, 31 and 23 kDa for heavy-chain fragments, and at 16 and 17.5 kDa for light chains [33]. Therefore, the band around 14–17 kDa observed in the present study could tentatively be associated with a myosin light chain or a related fragment [3,33].

Protein bands between 14 and 32 kDa may possibly originate from cuticular or enzymatic proteins, including chymotrypsin-like proteins with molecular weights around 24 kDa [43]. In TM, cuticular proteins have been reported mainly in the range of 14–30 kDa, which supports the assumption that bands in this region may partly originate from cuticular fractions [3]. The band around 20 kDa may additionally be associated with low-molecular-weight muscle-related proteins, such as calponin-like proteins or troponin C-like fractions, which have been reported in mealworm protein profiles [3]. The band around 45 kDa could be tentatively associated with enzyme fractions, such as melanization-inhibiting proteins with molecular weights of approximately 43 kDa [16], but may also correspond to actin-like or troponin T-like proteins, since actin-like proteins around 42 kDa and troponin T around 47 kDa have been reported in TM [3]. However, these assignments remain putative and would require protein identification analysis for confirmation.

The composition and intensity of TM bands may also be affected by the extraction and purification procedure. Pinel et al. [18] showed that mealworm protein concentrates obtained by isoelectric precipitation and ultrafiltration/diafiltration differed in protein content, structure, surface hydrophobicity, solubility and emulsifying properties.

In the ZM PI, several intense bands were also observed, including bands around 14 kDa, around 20 kDa, between 24 and 36 kDa, and between 45 and 66 kDa. As shown in Figure 1a, the most intense bands were located below 14 kDa, indicating the presence of low-molecular-weight protein fractions. These fractions may be of interest in relation to the bioactive potential of the ZM protein isolate, particularly as possible substrates for peptide release during subsequent enzymatic hydrolysis. However, their specific identity and contribution cannot be established from SDS-PAGE alone. The distribution of molecular weight fractions in the ZM PI was highly similar to that observed in the TM PI, which may again reflect their taxonomic relatedness. However, an additional fraction between 66 and 97 kDa was observed in the ZM PI, whereas this fraction was not detected in the TM PI. The presence of this additional 66–97 kDa fraction in the ZM PI may be related to species-specific differences in structural, storage or muscle-associated proteins. It may also reflect differences in protein solubility and recovery during alkaline extraction and isoelectric precipitation.

Recent studies on Zophobas morio proteins have shown that the extraction method can significantly influence protein recovery, molecular weight distribution, secondary structure and biological activity. Therefore, the differences observed between mealworm and superworm isolates may be attributed not only to taxonomy, but also to differences in tissue composition and extraction behaviour of particular protein fractions.

In the AD PI, five major bands were detected, with the most intense band located between 66 and 97 kDa. Similar to the mealworm and superworm PIs, the electrophoretic profile of the AD PI showed bands below 14 kDa and around 20 kDa. In addition, bands between 36 and 49 kDa and between 66 and 97 kDa were observed. The bands in the 36–49 kDa region may be tentatively associated with muscle-related or enzymatic proteins, such as actin-like proteins or arginine kinase-related fractions. This region is particularly important because several insect proteins within the range of approximately 30–45 kDa, including tropomyosin- and arginine kinase-related proteins, have been reported as relevant allergens in edible insects. Therefore, bands in this molecular weight range should be interpreted carefully, particularly when insect protein isolates are considered for food applications [3].

According to Lee et al. [44], protein fractions with molecular weights above 95 kDa may be associated with vitellogenin-like proteins. These proteins are important for oogenesis during certain insect developmental stages [45]. Recent literature also indicates that vitellogenin-like proteins in insects are generally associated with high-molecular-weight fractions, usually above 95 kDa [3]. In addition, high-molecular-weight structural and myofibrillar proteins may contribute to the electrophoretic profiles of edible insects. In TM, for example, α-actinin-like proteins around 107 kDa and myosin heavy-chain proteins above 200 kDa have been reported, indicating that high-molecular-weight muscle-associated proteins may be detected depending on extraction efficiency and gel resolution [3]. In addition to the above-mentioned protein fractions, high-molecular-weight structural proteins above 400 kDa may also be present in the cricket body. These proteins are involved in muscle structure, particularly in the formation of limb muscles, and have been described as kettin-like proteins [46]. Since the mealworm and superworm PIs were obtained from larval stages, the absence or lower intensity of such high-molecular-weight fractions may be related to differences in developmental stage and tissue composition.

Overall, the SDS-PAGE profiles indicated that the obtained PIs contained heterogeneous protein fractions distributed over a broad molecular weight range. Based on comparison with literature data, these bands may tentatively be associated with low-molecular-weight hemolymph or cuticular proteins, medium-molecular-weight enzymatic or muscle-associated proteins, and higher-molecular-weight structural or storage proteins. However, these assignments remain putative, since protein identification was not performed. The similarity between mealworm and superworm isolates suggests comparable electrophoretic patterns, possibly related to their taxonomic and developmental similarity, whereas the cricket isolate showed a distinct profile characterized by more pronounced high-molecular-weight bands. These differences may be relevant for understanding variations in functional properties among the three insect PIs, but further proteomic analysis would be required to confirm the identity of individual protein fractions.

### 3.4. Protein Solubility of PI

**Figure 2:**
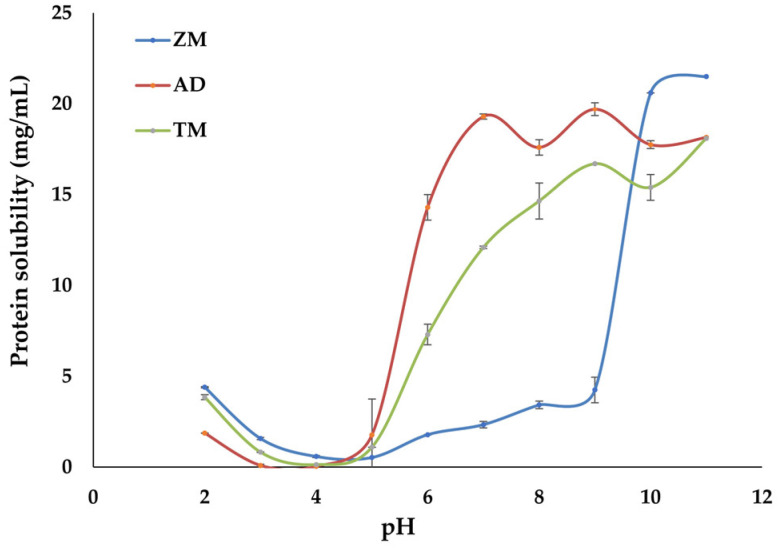
Protein solubility of insect PIs over a pH range from 2 to 11. Values are presented as mean ± standard deviation (SD). PI: protein isolate; TM: *Tenebrio molitor*; ZM: *Zophobas morio*; AD: *Acheta domesticus*.

The solubility of insect PIs was monitored over a pH range from 2 to 11, and the results are presented in Figure 2.

In acidic conditions, all three PIs showed relatively low solubility, whereas in alkaline conditions their solubility was considerably higher. The solubility curves of the TM and AD PIs were very similar, while the solubility curve of the ZM PI showed a somewhat different pattern. The most pronounced difference was observed in the alkaline region, where ZM proteins exhibited lower solubility at some pH values compared with mealworm and cricket proteins. The lowest solubility of all three PIs was observed in the pH range from 3 to 5. More specifically, the lowest solubility of mealworm and cricket proteins was recorded between pH 3 and pH 4, whereas the lowest solubility of ZM proteins was observed between pH 4 and pH 5. Similar results were reported by Bußler et al. [47], who found that the isoelectric point of most insect proteins is generally located in the pH range from 3 to 5. When all three PIs are considered together, the lowest protein solubility values were achieved at approximately pH 4. This decrease in solubility can be explained by the reduced net charge of proteins near their isoelectric point, which promotes protein–protein interactions, aggregation and precipitation. A significant increase in protein solubility was observed around pH 8 for AD PI (17.70 mg/mL) and TM PI (14.60 mg/mL). In the case of the ZM PIs, Figure 2 shows that a marked increase in solubility occurred at pH 10. The highest solubility values for all three PIs were achieved at pH 11, namely 21.50 mg/mL for ZM proteins, 18.15 mg/mL for AD proteins and 18.10 mg/mL for TM proteins. The increase in solubility under alkaline conditions is most likely related to the higher net negative charge of protein molecules, which increases electrostatic repulsion between protein chains and promotes protein hydration and dispersion. Similar behavior has been described for several edible insect protein ingredients, where the highest solubility was observed at alkaline pH values, particularly around pH 11 [3]. The obtained results are partly in agreement with those reported by Zhao et al. [48] and Zielińska et al. [15]. The main difference is related to protein solubility in acidic conditions, since previously published studies reported higher solubility of insect proteins in acidic media compared with the results obtained in the present research. The reduced solubility of proteins in acidic conditions may be associated with the technological processes used for the production of insect flours. Inactivation and drying of larvae and crickets, which involved the use of elevated temperatures, may have reduced protein extractability and solubility [5,17]. Although the inactivation of mealworm larvae and crickets was very short (30 s at 100 °C), it can be assumed that partial protein denaturation occurred, leading to reduced solubility [40]. Thermal treatment can induce protein unfolding, exposure of hydrophobic groups and subsequent aggregation, which may reduce the ability of proteins to interact with water. In addition to thermal treatment, the isolation method itself may strongly influence the solubility of insect protein isolates. Recent research on TM concentrates showed that the processing method affects protein structure, denaturation, surface hydrophobicity and techno-functional properties. Pinel et al. [18] reported that mealworm protein concentrates obtained by ultrafiltration/diafiltration had higher solubility and better emulsifying properties at neutral pH than concentrates obtained by isoelectric precipitation. This indicates that protein solubility should not be interpreted only as a species-dependent property, but also as a consequence of extraction, purification and precipitation conditions. Nevertheless, it should be emphasized that the solubility of all three PIs was very good in alkaline conditions and can be compared with the solubility of proteins from some legumes [49,50]. In neutral conditions, insect proteins generally show lower solubility compared with some plant proteins and legume proteins [51]. This may be explained by the type and composition of proteins present in insect PIs. Insect PIs may contain a higher proportion of proteins rich in non-polar amino acid residues and structural proteins, whereas plant proteins often contain albumins and globulins, which are more soluble in water and salt solutions [5,52]. Furthermore, the presence of chitin, lipids, minerals or protein–chitin complexes in insect-derived ingredients may additionally affect protein extractability and solubility. Overall, the obtained solubility profiles indicate that the three insect PIs have strongly pH-dependent behavior, with minimum solubility near the isoelectric region and maximum solubility under alkaline conditions. Such behavior is important for their potential application in food systems, since higher protein solubility can improve emulsifying and foaming properties, while lower solubility near the isoelectric point may limit their use in acidic food products unless additional treatments, such as enzymatic hydrolysis, pH-shifting, ultrasound treatment or modification of extraction conditions, are applied.

### 3.5. Water and Oil Holding Capacities of PI

As shown in Table 3, the highest oil-holding capacity was observed for the ZM PI with a value of 0.94 g/g. The lowest oil-binding capacity was recorded for the AD PIs, 0.51 g/g, whereas the TM PIs showed an intermediate value of 0.61 g/g. The obtained oil-binding capacity values were lower than those reported by Stone et al. [5], who observed oil-binding capacities above 1 g/g for cricket and superworm PIs. However, it should be noted that their results were expressed on a dry matter basis, which may partly explain the differences and indicates that the values obtained in the present study are still comparable. OHC capacity is mainly influenced by the ability of oil to penetrate the protein matrix, as well as by the number and accessibility of non-polar amino acid side chains within the protein structure [27]. In general, proteins with a higher proportion of exposed hydrophobic regions are expected to retain more oil, which can improve texture, mouthfeel and flavour retention in food systems. Several studies have shown that enzymatic hydrolysis may improve oil-binding capacity. During hydrolysis, partial unfolding of the protein structure occurs, exposing hydrophobic amino acid residues that are usually located inside the native protein structure. These exposed hydrophobic groups can enhance protein–lipid interactions and increase oil retention [53,54]. This is particularly relevant for insect proteins, since enzymatic hydrolysis has also been reported to improve some techno-functional properties, especially solubility and emulsifying behaviour, depending on the enzyme used and the degree of hydrolysis. The oil-binding capacity obtained for the ZM PI was higher than values reported for some plant protein ingredients, including soybean (0.84 g/g) and lentil proteins (0.93 g/g) [55]. Therefore, ZM PI may have potential for application in food formulations in which oil retention and flavour binding are desirable. These properties may be useful in meat analogues, emulsified meat products, bakery products and other systems in which proteins contribute to texture, juiciness and lipid stabilization. WHC of the analysed insect PIs ranged from 1.32 to 2.01 g/g. The highest WHC was recorded for the ZM PI, 2.01 g/g, whereas the lowest value was observed for the TM isolate, 1.32 g/g. The AD PI showed an intermediate water-binding capacity of 1.51 g/g. The obtained values are in agreement with previously published data for edible insect protein ingredients [5,15], while they are somewhat higher than the value reported by Bußler et al. [47], who observed a water-binding capacity of approximately 0.80 g/g. Differences in WHC among studies may be attributed to insect species, developmental stage, protein composition, defatting procedure, drying conditions and extraction method [56]. WHC is generally associated with the availability of hydrophilic amino acid residues and polar groups, which can interact with water through hydrogen bonding and electrostatic interactions [4]. In addition, protein denaturation may expose previously buried polar groups, thereby improving water retention; however, excessive denaturation and aggregation may have the opposite effect and reduce the accessibility of water-binding sites. Compared with some commercially available plant proteins, WHC values obtained for all three insect PIs indicate that these ingredients may be competitive with conventional protein sources [15]. WHC is an important functional property because it contributes to viscosity, texture, juiciness and product yield, especially in comminuted meat products, meat analogues, bakery products and protein-enriched foods. In contrast to OHC, the literature data suggest that enzymatic hydrolysis does not always improve water-binding capacity and that its effect depends on the extent of hydrolysis and the balance between newly exposed hydrophilic groups and reduced molecular size [57]. Thermal treatments may also affect water- and oil-binding capacities differently. Moderate heating can induce partial unfolding and expose hydrophobic regions, which may increase OHC, whereas severe heating may promote aggregation and reduce the availability of hydrophilic groups, thereby negatively affecting water retention [40]. Overall, based on the obtained WHC and OHC results, all three insect PIs showed functional potential for use as alternative protein ingredients in food systems. Among the analysed samples, the superworm PI exhibited the most favourable binding properties, with the highest values for both OHC and WHC. These results suggest that insect PIs, particularly ZM, may be suitable for food applications in which water retention, oil binding, texture improvement and formulation stability are important.

### 3.6. Antioxidative Activity of PI

The antioxidant activity of insect PIs was evaluated using the ABTS assay, which measures the ability of samples to scavenge ABTS•+ radical cations. The antioxidant activity results are presented in Table 3. The antioxidant potential was expressed as the IC_50_ value, defined as the PI concentration required to neutralize 50% of ABTS•+ radicals. Lower IC_50_ values indicate stronger antioxidant activity. The IC_50_ values ranged from 0.108 to 0.22 mg/mL, indicating that all three insect PIs exhibited notable ABTS radical-scavenging activity. Although the ZM PI showed the numerically lowest IC_50_ value, the differences among the samples were not statistically significant (*p* > 0.05). Therefore, the antioxidant activity of the three PIs can be considered comparable under the applied experimental conditions. The observed antioxidant activity may be partly related to the presence of low-molecular-weight protein fractions below 14 kDa, as shown by SDS-PAGE. Such fractions may include short peptides or easily hydrolysable proteins that can contribute to radical-scavenging activity. However, since peptide profiling was not performed, this relationship should be considered only as a possible explanation rather than a confirmed mechanism. Antioxidant activity depends not only on molecular weight, but also on peptide sequence, amino acid composition, hydrophobicity and the presence of amino acid residues capable of donating electrons or hydrogen atoms. Compared with insect PHs reported in the literature, the antioxidant activity of the non-hydrolysed isolates may be lower, which is expected because enzymatic hydrolysis promotes the release of shorter bioactive peptides and increases the accessibility of antioxidant-active amino acid residues. Recent reviews confirm that insect-derived peptides obtained by enzymatic hydrolysis often show stronger antioxidant activity than native proteins or PIs, depending on enzyme type, degree of hydrolysis and peptide profile [3]. Overall, PIs from TM, ZM and AD showed comparable antioxidant potential.

### 3.7. Antimicrobial Activity of PI

The obtained results, which are presented in Tables S1 and S2 indicated that none of the PIs exhibited antimicrobial activity against *E. coli* or *S. epidermidis*. Based on the electrophoretic profile of PIs, the presence of low-molecular-weight protein fractions was observed; however, this method does not provide quantitative insight into their abundance. Therefore, it may be assumed that, although present, these low-molecular-weight protein fractions were not present in sufficient amounts to exert antimicrobial activity.

### 3.8. Protein Hydrolysates

#### 3.8.1. Protein Hydrolysis and SDS Gel Electrophoresis

**Figure 3:**
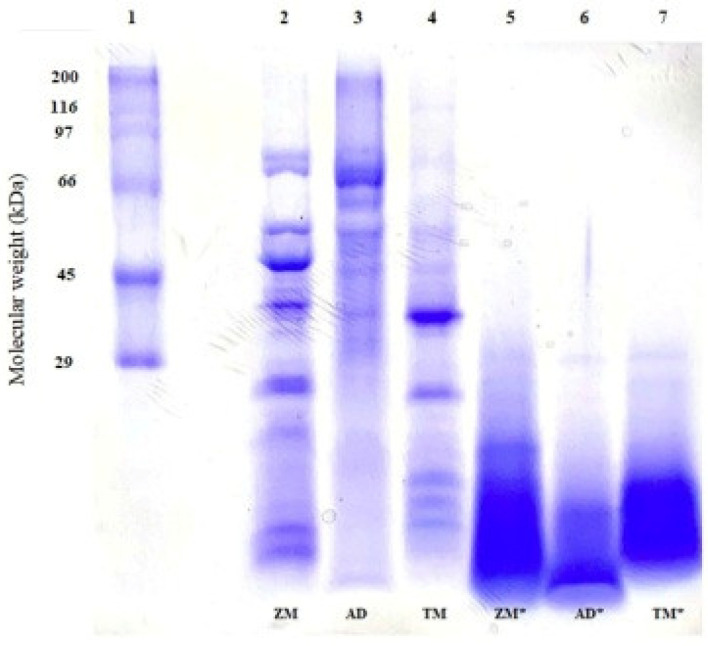
Electrophoretic profiles of protein isolates and protein hydrolysates obtained from *Zophobas morio* (ZM), *Acheta domesticus* (AD), and *Tenebrio molitor* (TM) in the molecular weight range of 29–200 kDa. Lane 1: molecular weight standard; lanes 2–4: protein isolates (PI) of ZM, AD and TM, respectively; lanes 5–7: corresponding protein hydrolysates (PH) of ZM*, AD* and TM*, respectively. The asterisk (*) denotes protein hydrolysate samples.

The electrophoretic profiles of the PIs and PHs obtained from all three insect species are shown in Figure 3. As expected, the most intense bands in the PHs were observed below 29 kDa, indicating enzymatic degradation of high-molecular-weight protein fractions that were present in the corresponding PIs. In contrast to the PI, high-molecular-weight protein fractions were not detected in the hydrolysates, confirming the successful enzymatic hydrolysis of insect proteins. These results are in agreement with the findings of Hall et al. [58] and Yoon et al. [59], who reported that after Alcalase hydrolysis of cricket and mealworm proteins, the resulting protein fractions were predominantly below 14 kDa. Based on the obtained results, it can be concluded that a low enzyme concentration of 0.5%, corresponding to an enzyme-to-substrate ratio of 1:200, was sufficient to hydrolyse high-molecular-weight protein fractions and generate low-molecular-weight protein/peptide fractions. Although such fractions are frequently associated with bioactive potential, their specific contribution in the present study cannot be confirmed based on SDS-PAGE alone.

**Figure 4:**
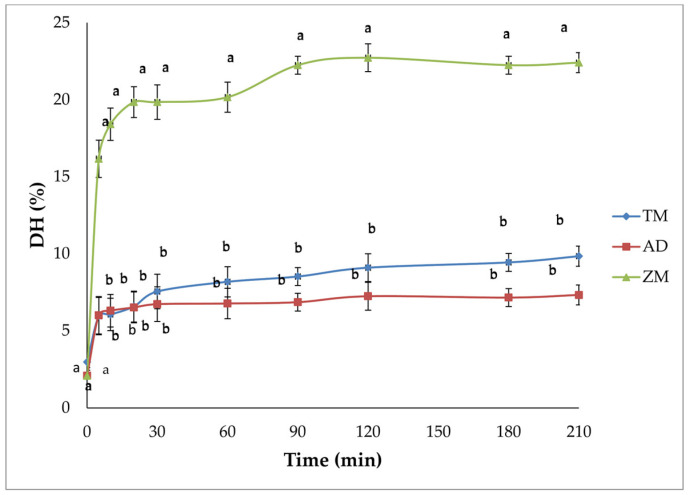
Degree of hydrolysis (DH) of *Tenebrio molitor* (TM), *Acheta domesticus* (AD) and *Zophobas morio* (ZM) protein isolates during Alcalase hydrolysis. Values are presented as mean ± standard deviation, n = 3. Different letters next to the data points indicate statistically significant differences at *p* ≤ 0.05.

Enzymatic hydrolysis was performed using Alcalase under controlled conditions, with the aim of obtaining hydrolysates enriched in low-molecular-weight protein and peptide fractions for further evaluation of their antibacterial activity. PIs were dissolved in glycine buffer at pH 9 in order to ensure satisfactory protein solubility, suitable conditions for Alcalase activity, and minimal influence of pH on bacterial growth. The time course of enzymatic hydrolysis for all three PIs is shown in Figure 4.

The hydrolysis curves showed a typical enzymatic reaction pattern, characterized by an initial rapid increase in the degree of hydrolysis (DH), followed by a slower reaction phase and a final plateau. Similar hydrolysis profiles have previously been reported for soybean proteins and oil pumpkin proteins [28,60]. At the initial time point, the DH of the control samples was approximately 2%, which may be related to partial protein modification during previous thermal processing of insect flours. The DH values of all hydrolysates are summarized in Table 4. The ZM PI exhibited the highest DH (22.4%), which was significantly higher than the values obtained for the TM and AD PIs (9.83% and 7.32%, respectively). No statistically significant difference was observed between the TM and AD PIs. The higher DH of the ZM PI may be associated with its lower protein concentration and different protein accessibility under the applied hydrolysis conditions. At pH 9, the protein concentration of ZM was 6.25 mg/mL, while TM and AD isolates showed higher concentrations, 17.5 and 23.5 mg/mL, respectively. These differences suggest that protein solubility, substrate concentration and protein structure influenced the efficiency of Alcalase hydrolysis. The obtained DH values are in accordance with literature data showing that DH strongly depends on protein source, enzyme type, enzyme/substrate ratio, pH, temperature and initial protein structure. Higher DH values reported in some previous studies may be explained by the use of different enzymes, such as pepsin, or higher enzyme/substrate ratios [58,61]. Overall, the results indicate that ZM PI was the most susceptible to Alcalase hydrolysis under the applied conditions, while mealworm and cricket isolates showed lower but comparable hydrolysis levels.

Obtained PHs showed ABTS radical-scavenging activity, with the IC_50_ values following the order ZM < TM < AD. The lowest estimated IC_50_ value was observed for the ZM hydrolysate (0.055 mg/mL), followed by TM (0.105 mg/mL) and AD (0.145 mg/mL), indicating the highest expected antioxidant potential of the ZM hydrolysate. This trend may be related to the higher DH of ZM, which could indicate more extensive protein degradation and formation of lower-molecular-weight protein/peptide fractions with potential relevance for radical-scavenging activity. In addition to DH and molecular size, the amino acid composition may also have contributed to the observed antioxidant activity. In particular, ZM PI contained the highest levels of tyrosine and histidine among the analysed samples, which may support the stronger ABTS radical-scavenging activity observed for the corresponding hydrolysate. Tyrosine and histidine are known to contribute to antioxidant activity through their ability to participate in hydrogen or electron donation reactions. Nevertheless, in the absence of peptide identification, this relationship should be interpreted as a possible contributing factor rather than a confirmed mechanism.

#### 3.8.2. Visual Evaluation of Antimicrobial Activity

The first assay performed was based on visual evaluation, which provided an initial indication of the antimicrobial potential of the insect protein hydrolysates. Based on this method, the inhibitory effect of PHs, expressed as the minimum inhibitory concentration (MIC), was determined as the lowest concentration preventing the reduction of resazurin from its oxidized to reduced form [30]. In the presence of bacterial growth, resazurin changed colour to pink, whereas in the absence of bacterial growth it remained blue.

**Figure 5:**
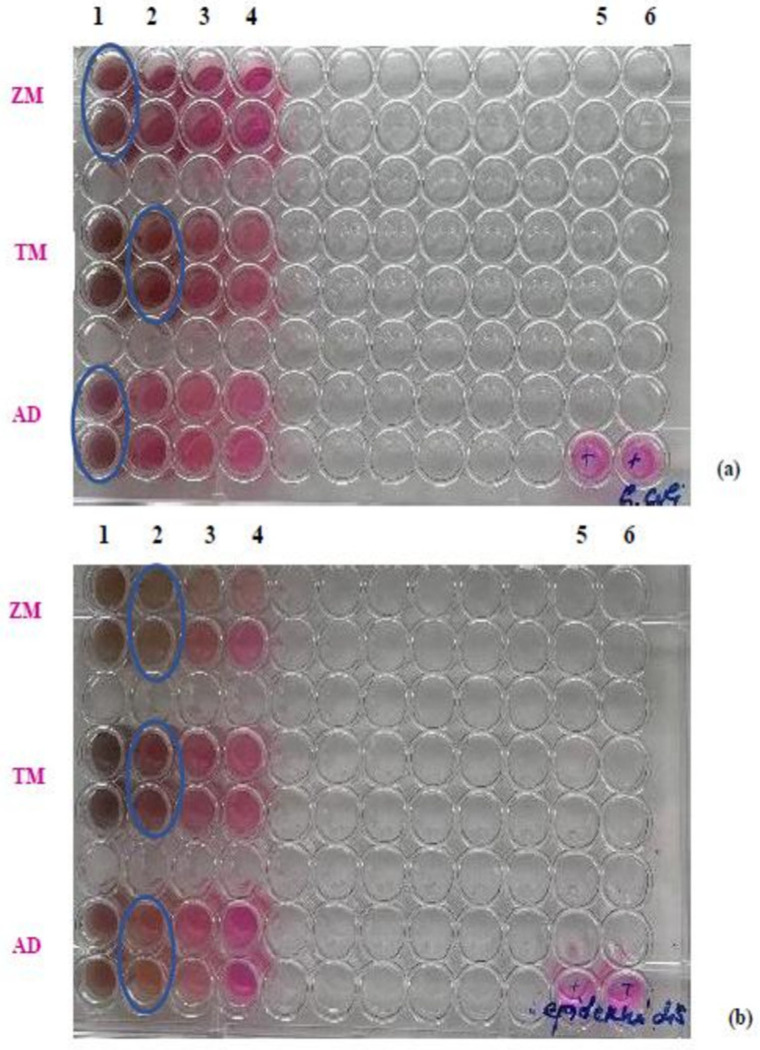
Visual assessment of antimicrobial activity of protein hydrolysates from *Zophobas morio* (ZM), *Tenebrio molitor* (TM) and *Acheta domesticus* (AD) against (**a**) *Escherichia coli* and (**b**) *Staphylococcus epidermidis* using the microdilution assay. Columns 1–4 represent decreasing hydrolysate concentrations, as shown in Table 5. Columns 5 and 6 represent controls. Dark blue or purple wells indicate the absence of detectable bacterial growth, while pink wells indicate bacterial growth due to the reduction of resazurin. Wells enclosed by blue outlines indicate the lowest hydrolysate concentration at which no color change associated with bacterial growth was observed and were therefore used for MIC determination.

Visual observation of the microtiter plates shown in Figure 5 indicated that concentration 1 of all PHs exhibited a visible inhibitory effect against both *E. coli* and *S. epidermidis*. At concentration 2, the inhibitory effect of mealworm PH was observed against *E. coli*, while the PHs obtained from superworm and mealworm inhibited the growth of *S. epidermidis*. The concentrations of PHs used in the microtiter plates are presented in Table 5.

The obtained results indicated that, in the case of *E. coli*, the MIC value of ZM PH was 5.68 mg/mL, whereas the MIC value of mealworm PH was 7.95 mg/mL. The weakest antimicrobial effect against *E. coli* was observed for AD PH, with an MIC value of 21.36 mg/mL. Insect PHs showed stronger antimicrobial activity against *S. epidermidis*. The MIC values against this bacterium were 2.84 mg/mL for ZM PH, 7.95 mg/mL for mealworm PH and 10.67 mg/mL for cricket PH. Among all investigated hydrolysates, ZM PH showed the strongest antimicrobial effect. The pronounced activity of ZM PH at relatively low concentrations may be associated with its highest DH (22.40%), suggesting more extensive protein hydrolysis and the formation of lower-molecular-weight protein and peptide fractions. However, in the absence of peptide identification, their specific contribution to the observed antimicrobial activity remains to be confirmed.

Insects have been used for centuries in traditional Chinese medicine for the treatment of colds, cough, fever, childhood epilepsy, rubella, tetanus and other diseases [62]. In this context, increasing attention has recently been directed towards antimicrobial peptides (AMPs) of insect origin and their antibacterial properties [63]. Ma et al. [64] investigated the antimicrobial activity of different insect extracts against highly resistant bacteria, including *Staphylococcus aureus* and *Mycobacterium tuberculosis*. Their results showed that cricket extracts obtained using ethyl acetate and n-butanol inhibited the growth of both bacterial strains, with MIC values of 100 mg/mL. Similarly, Flores et al. [65] reported that mealworm protein hydrolysates showed significant antibacterial activity against both Gram-negative bacteria, such as *Proteus vulgaris* and *Shigella flexneri*, and Gram-positive bacteria, such as *Bacillus* spp. These findings are in agreement with the results obtained in the present study.

#### 3.8.3. Reduction in Bacterial Growth

**Figure 6:**
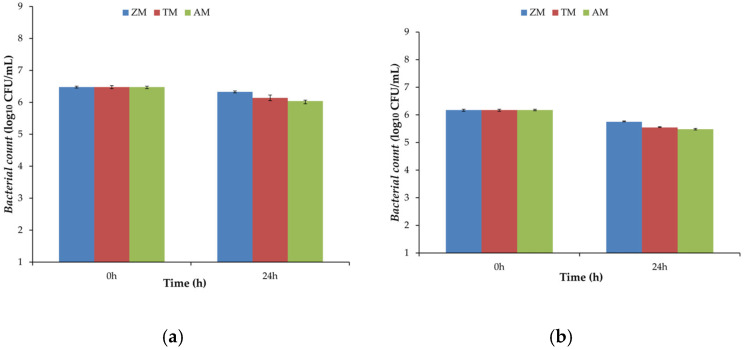
Effect of PHs from *Zophobas morio* (ZM), *Tenebrio molitor* (TM) and *Acheta domesticus* (AD) on the growth of (**a**) *Escherichia coli* and (**b**) *Staphylococcus epidermidis* after 24 h of incubation. Results are expressed as log_10_ CFU/mL and presented as mean ± standard deviation, n = 3.

After 24 h of incubation, a reduction in the number of *E. coli* colonies was observed in all Petri dishes containing insect PHs (Figure 6). Plates without PHs showed denser bacterial colonies compared with plates containing PHs. These results indicate that all investigated insect PHs exerted an inhibitory effect on the growth of *E. coli*.

*E. coli* naturally inhabits the intestinal tract of humans and animals and generally does not cause disease. However, certain strains of *E. coli* are able to produce Shiga toxin and may cause endemic diseases in piglets and dysentery in calves on farms [66]. In humans, food contaminated with pathogenic *E. coli* can cause bloody diarrhea and urinary tract infections [67]. Therefore, preventing contamination with this bacterium is highly important in both food and feed production.

Figure 6 shows the reduction in *E. coli* growth on nutrient agar. The reductions were expressed as Δlog_10_ CFU/mL. Based on the mean values, AD PH produced the largest decrease in viable *E. coli* counts, followed by TM PH and ZM PH, with reductions of 0.44 Δlog_10_ CFU/mL, 0.34 Δlog_10_ CFU/mL, and 0.15 Δlog_10_ CFU/mL, respectively. The observed differences may have been partly influenced by the different protein concentrations used in the time-kill assay, which were highest for AD PH (23.50 mg/mL), followed by TM PH (17.50 mg/mL) and ZM PH (6.25 mg/mL). Relative to the MIC values against *E. coli*, these concentrations corresponded to approximately 1.1× MIC for ZM PH, 2.2× MIC for TM PH, and 1.1× MIC for AD PH. Consequently, direct quantitative comparison among the three hydrolysates is limited, particularly because TM PH was evaluated at a higher MIC multiple than ZM and AD PH.

Several decades ago, it was demonstrated that the insect immune system contains cecropin, a peptide involved in protection against Gram-negative bacteria such as *E. coli* [68,69]. In addition, attacin, a peptide with a molecular weight between 20 and 25 kDa, has been isolated from insects and shown to possess strong antimicrobial activity against Gram-negative bacteria [70]. However, the presence of these or any other specific antimicrobial peptides was not confirmed in the hydrolysates analysed in the present study. The electrophoretic profile only indicated the presence of protein or peptide fractions below 29 kDa, but molecular weight alone is insufficient for their identification. Further LC-MS/MS analysis, peptide purification, and activity-guided fractionation would be required to identify the active compounds responsible for the inhibitory effect against *E. coli*.

*S. epidermidis* is a bacterium naturally present on human skin and the nasal mucosa. Its pathogenic potential is most often expressed in hospital environments, where it forms biofilms on plastic medical equipment, including catheters and other medical devices [71]. On farms, *S. epidermidis* can cause mastitis in lactating cows, which represents a serious problem in milk production and often requires antibiotic treatment [72]. Previous studies have also shown that this bacterium may be associated with various disorders related to kidney, liver and intestinal damage in humans and animals [73,74]. These findings highlight the need to prevent the spread of this bacterium and to develop new, efficient and economically acceptable control strategies.

The reduction in the growth of the Gram-positive bacterium *S. epidermidis* was more pronounced after treatment with all three PHs (Figure 5 and Figure 6). According to the obtained results, the largest decrease in viable *S. epidermidis* counts was observed for AD PH (0.69 Δlog_10_ CFU/mL), followed by TM PH and ZM PH, with approximate reductions of 0.63 and 0.42 Δlog_10_ CFU/mL, respectively. Previous studies have identified several AMPs with activity against Gram-positive bacteria, including sarcotoxin, hyphancin and enbocin [75]. Since the AMPs present in the hydrolysates were not identified in the present study, it can only be assumed that similar peptide fractions may have contributed to the reduction in *S*. *epidermidis* growth. In contrast to the assay against *E. coli*, all three PHs were tested against *S. epidermidis* at approximately the same relative concentration of 2.2× MIC. Therefore, comparison of their effects against *S. epidermidis* is more appropriate than comparison of their effects against *E. coli*.

All three PHs produced greater Δlog_10_ CFU/mL reductions against *S. epidermidis* than against *E. coli* under the applied experimental conditions. However, comparison between the two bacterial species should take into account that the hydrolysates were not consistently tested at identical MIC multiples in the *E. coli* assay. Therefore, the observed differences cannot be attributed solely to the bacterial Gram type or to the intrinsic activity of the hydrolysates. The discrepancy between the MIC and time-kill results is not necessarily contradictory because these assays evaluate different antimicrobial endpoints. MIC represents the lowest concentration preventing detectable bacterial growth in a microdilution assay, whereas the time-kill assay measures changes in viable bacterial counts over a defined period. A hydrolysate with a lower MIC may therefore not necessarily produce the largest reduction in viable counts at a single tested concentration. The antimicrobial activity may have been influenced by the tested protein concentration, the concentration relative to the MIC, and the structural properties of the generated protein and peptide fractions, including molecular size, amino acid composition, net charge, hydrophobicity, and potential amphipathic character. Smaller and positively charged hydrophobic fractions may interact more efficiently with bacterial cell envelopes. However, since peptide identification was not performed, the active structures and their mechanisms of action remain unknown. The increasing resistance of bacteria to conventional antibiotics represents one of the major challenges in food safety, animal production and human health. *S. aureus*, for example, is a well-known pathogen capable of causing various infections and diseases in humans and animals [76,77,78], and it has developed resistance to many commercially available antibiotics [64]. Therefore, there is growing interest in identifying natural antimicrobial compounds that could serve as alternatives or complementary agents to synthetic antibiotics. Based on the results obtained in this study, PHs from mealworm, superworm and cricket showed potential antimicrobial activity against both Gram-negative and Gram-positive bacteria. These findings suggest that insect PHs could be considered promising ingredients for future applications in the food, feed, pharmaceutical and cosmetic industries, not only as nutritionally valuable protein-rich materials, but also as potential sources of AMP. Nevertheless, further studies are necessary to identify the active peptide fractions, determine their mechanisms of action, evaluate their safety and confirm their effectiveness in real food and feed systems.

An important limitation of this study is that peptide sequence identification was not performed. Therefore, the observed antioxidant and antimicrobial activities cannot be attributed to specific peptide sequences or individual bioactive compounds. Although SDS-PAGE indicated the formation of lower-molecular-weight fractions after enzymatic hydrolysis, further LC-MS/MS-based peptide profiling, peptide purification and activity-guided fractionation are required to identify the active fractions and clarify their mechanisms of action.

## 4. Conclusions

This study demonstrated that PIs and PHs from TM, ZM and AD represent promising sources of functional and bioactive ingredients. All PIs showed notable antioxidant activity in the ABTS assay, which may be partly related to the presence of low-molecular-weight protein fractions observed by SDS-PAGE. The non-hydrolysed PIs did not show antimicrobial activity against *E. coli* and *S. epidermidis* under the applied conditions. However, enzymatic hydrolysis with Alcalase enhanced their bioactive potential and led to the formation of lower-molecular-weight fractions. The highest DH was obtained for the ZM PI, while lower values were observed for TM and AD. All obtained PHs showed inhibitory effects against both tested bacterial strains, with stronger activity generally observed against the Gram-positive bacterium *S. epidermidis*. Among the tested samples, AD hydrolysate showed the highest reduction in bacterial growth, while ZM hydrolysate exhibited the strongest antioxidant potential. Overall, the results indicate that edible insect PIs and PHs may have potential applications as natural antioxidant and antimicrobial ingredients in food, feed, pharmaceutical and cosmetic systems. However, further studies addressing allergenicity, toxicity, digestibility, and proteomic identification of bioactive peptides are required to better understand their mechanisms of action and to support their safe commercial application.

## Figures and Tables

**Table 1:** Chemical composition of insect PI obtained after double alkaline extraction.

Protein Isolate	TM	ZM	AD
Protein content (cf 5.6) (%)	74.98 ± 1.17 ^a^	65.29 ± 0.81 ^c^	68.88 ± 1.11 ^b^
Moisture (%)	4.25 ± 0.31 ^a^	4.75 ± 0.15 ^ab^	5.1 ± 0.17 ^b^
Fat (%)	1.25 ± 0.11 ^a^	1.17 ± 0.12 ^a^	1.41 ± 0.27 ^a^
Ash (%)	3.15 ± 0.31 ^ab^	2.45 ± 0.19 ^b^	4.1 ± 0.71 ^a^

Values are presented as mean ± standard deviation. Different superscript letters within the same row indicate statistically significant differences according to Tukey’s HSD test at *p* < 0.05. PI: protein isolate; TM: *Tenebrio molitor*; ZM: *Zophobas morio*; AD: *Acheta domesticus*; cf: conversion factor.

**Table 2:** Amino acid composition of insect PI expressed per 100 g of protein (%).

Amino Acid	TM	ZM	AD
Aspartic acid	6.86 ± 0.31 ^b^	8.65 ± 0.17 ^a^	5.59 ± 0.71 ^c^
Tyrosine	8.25 ± 0.19 ^b^	10.20 ± 0.31 ^a^	2.62 ± 0.14 ^c^
Arginine	4.34 ± 0.21 ^b^	5.44 ± 0.27 ^a^	4.09 ± 0.15 ^b^
Serine	3.29 ± 0.17 ^a^	3.51 ± 0.12 ^a^	2.05 ± 0.11 ^b^
Glutamic acid	6.49 ± 0.31 ^b^	7.96 ± 0.33 ^a^	4.61 ± 0.21 ^c^
Proline	0.07 ± 0.01 ^b^	0.24 ± 0.10 ^b^	2.83 ± 0.11 ^a^
Glycine	4.78 ± 0.13 ^a^	4.52 ± 0.17 ^a^	3.16 ± 0.17 ^b^
Alanine	3.33 ± 0.17 ^b^	3.88 ± 0.21 ^a^	3.13 ± 0.21 ^b^
Cystine	0.13 ± 0.07 ^a^	0.15 ± 0.04 ^a^	0.12 ± 0.05 ^a^
Valine	4.36 ± 0.31 ^b^	5.61 ± 0.37 ^a^	2.64 ± 0.17 ^c^
Methionine	0.91 ± 0.07 ^b^	1.60 ± 0.01 ^a^	1.08 ± 0.13 ^b^
Isoleucine	4.15 ± 0.31 ^b^	5.44 ± 0.07 ^a^	2.39 ± 0.15 ^c^
Leucine	5.25 ± 0.21 ^b^	6.45 ± 0.21 ^a^	3.23 ± 0.19 ^c^
Threonine	2.91 ± 0.17 ^b^	3.97 ± 0.13 ^a^	2.03 ± 0.12 ^c^
Phenylalanine	ND	ND	ND
Histidine	1.77 ± 0.18 ^b^	3.09 ± 0.05 ^a^	1.11 ± 0.15 ^c^
Lysine	3.88 ± 0.27 ^b^	5.83 ± 0.07 ^a^	4.11 ± 0.27 ^b^
ƩNEAA	37.54	44.56	28.21
ƩEAA	23.24	31.99	16.59
TAA	60.78	76.55	44.80

Values are expressed as mean ± standard deviation (SD). Different superscript letters within the same row indicate statistically significant differences among samples according to one-way ANOVA followed by Tukey’s post hoc test (*p* < 0.05). PI: protein isolate; ND: not detected; TM: *Tenebrio molitor*; ZM: *Zophobas morio*; AD: *Acheta domesticus*; ΣNEAA: total non-essential amino acids; ΣEAA: total essential amino acids; TAA: total amino acids.

**Table 3:** Functional properties and antioxidant activity of insect protein isolates.

Functional Property	TM	ZM	AD
OHC (g oil/g sample)	0.61 ± 0.07 ^b^	0.94 ± 0.05 ^a^	0.51 ± 0.02 ^b^
WHC (g water/g sample)	1.32 ± 0.05 ^c^	2.01 ± 0.03 ^a^	1.51 ± 0.02 ^b^
AA IC_50_ (mg/mL)	0.20±0.11 ^a^	0.108 ± 0.08 ^a^	0.22 ± 0.12 ^a^

Values are expressed as mean ± standard deviation (SD). Different superscript letters within the same row indicate statistically significant differences among samples according to one-way ANOVA followed by Tukey’s post hoc test (*p* < 0.05). TM: *Tenebrio molitor*, ZM: *Zophobas morio*, AD: *Acheta domesticus*, OHC: oil-holding capacity; WHC: water-holding capacity; AA IC_50_, antioxidant activity expressed as the concentration required to inhibit 50% of activity.

**Table 4:** Degree of hydrolysis and ABTS radical-scavenging activity of protein hydrolysates (PHs) from *Zophobas morio* (ZM), *Tenebrio molitor* (TM) and *Acheta domesticus* (AD).

Protein Hydrolysate	DH (%)	IC_50_ (mg/mL)
ZM	22.4 ± 1.32 ^a^	0.055 ± 0.006 ^a^
TM	9.83 ± 1.72 ^b^	0.105 ± 0.011 ^b^
AD	7.32 ± 1.72 ^b^	0.145 ± 0.014 ^c^

Results are presented as mean ± standard deviation (n = 3). Different superscript letters within the same column indicate statistically significant differences.

**Table 5:** Degree of hydrolysis (DH) of protein hydrolysates from *Zophobas morio* (ZM), *Tenebrio molitor* (TM) and *Acheta domesticus* (AD) and their concentrations used in wells 1–4 of the microdilution assay.

PH	DH (%)	Hydrolysate Concentration (mg/mL)			
		1	2	3	4
**ZM**	22.4 ± 1.32	5.68	2.84	1.42	0.71
**TM**	9.83 ± 1.72	15.91	7.95	3.98	1.99
**AD**	7.32 ± 1.72	21.36	10.67	5.34	2.67

## Data Availability

The original contributions presented in this study are included in the article and Supplementary Material. Further inquiries can be directed to the corresponding author.

## Supplementary Materials

The following supporting information can be downloaded at: https://www.mdpi.com/article/10.3390/foods15152729/s1, Table S1. Effect of protein isolates (PIs) from *Zophobas morio* (ZM), *Tenebrio molitor* (TM) and *Acheta domesticus* (AD) on the growth of *Escherichia coli* after 24 h of incubation. Table S2. Effect of protein isolates (PIs) from *Zophobas morio* (ZM), *Tenebrio molitor* (TM) and *Acheta domesticus* (AD) on the growth of *Staphylococcus epidermidis* after 24 h of incubation.

## Abbreviations

The following abbreviations are used in this manuscript:

TM	*Tenebrio molitor*
ZM	*Zophobas morio*
AD	*Acheta domesticus*
PI	Protein isolate
PH	Protein hydrolysate
SDS-PAGE	Sodium dodecyl sulfate–polyacrylamide gel electrophoresis
WHC	Water-holding capacity
OHC	Oil-holding capacity
ABTS	2,2′-azinobis(3-ethylbenzothiazoline-6-sulfonic acid)
ABTS•+	ABTS radical cation
IC_50_	Concentration required to inhibit/scavenge 50% of activity
MIC	Minimum inhibitory concentration
DH	Degree of hydrolysis
TCA	Trichloroacetic acid
MHB	Mueller–Hinton broth
MHA	Mueller–Hinton agar
NA	Nutrient agar
PBS	Phosphate-buffered saline
BSA	Bovine serum albumin
E/S	Enzyme-to-substrate ratio
CLSI	Clinical and Laboratory Standards Institute
AOAC	Association of Official Analytical Chemists
CFU	Colony-forming units
TAA	Total amino acids
DPP-IV/DPP-4	Dipeptidyl peptidase IV
ACE	Angiotensin-converting enzyme
DIAAS	Digestible Indispensable Amino Acid Score
AMP	Antimicrobial peptide
HCl	Hydrochloric acid
NaOH	Sodium hydroxide
SDS	Sodium dodecyl sulfate
PTFE	Polytetrafluoroethylene
