# Supplementary material for: Bioactive and Functional Properties of Protein Isolates and Hydrolysates from Edible Insects
Source: Foods. 2026 Aug 3;15(15):2729. doi: 10.3390/foods15152729 (PMC13464921; doi:10.3390/foods15152729)
Supplement: Supplementary file 1 [file foods-15-02729-s001.zip › foods-4424178-supplementary.pdf]

Supplementary Material

Table S1. Effect of protein isolates (PIs) from *Zophobas morio* (ZM), *Tenebrio molitor* (TM) and *Acheta domesticus* (AD) on the growth of *Escherichia coli* after 24 h of incubation.

Treatment	0 h (log_{10}CFU/mL)	24 h (log_{10}CFU/mL)
Control	6.48±0.03	8.45±0.03
ZM PI	6.48±0.03	8.44±0.04
TM PI	6.48±0.03	8.42±0.04
AD PI	6.48±0.03	8.45±0.04

Results are expressed as log_{10} CFU/mL and presented as mean ± standard deviation (n = 3).

Table S2. Effect of protein isolates (PIs) from *Zophobas morio* (ZM), *Tenebrio molitor* (TM) and *Acheta domesticus* (AD) on the growth of *Staphylococcus epidermidis* after 24 h of incubation.

Treatment	0 h (log_{10}CFU/mL)	24 h (log_{10}CFU/mL)
Control	6.18±0.03	8.20±0.04
ZM PI	6.18±0.03	8.18±0.05
TM PI	6.18±0.03	8.16±0.04
AD PI	6.18±0.03	8.21±0.03

Results are expressed as log_{10} CFU/mL and presented as mean ± standard deviation (n = 3).
